# Can nutritional grouping lower feed costs and enteric methane when diets are optimized with modest ingredient changes?

**DOI:** 10.3168/jdsc.2025-0919

**Published:** 2026-02-19

**Authors:** Yijing Gong, Chiung-Lun Huang, Victor E. Cabrera

**Affiliations:** Department of Animal and Dairy Sciences, University of Wisconsin–Madison, Madison, WI 53706

## Abstract

Nutritional grouping (NG), where cows are fed in groups based on similar nutrient requirements, has been used as a strategy to improve the precision of nutrition delivery and thus economic returns. Enteric methane, one of the largest on-farm sources of greenhouse gas emissions, is strongly influenced by diet formulation. With growing concerns about climate change, it is important to evaluate whether NG can also help reduce enteric methane emissions. To address this, we developed an open-source linear optimization model to assess the impact of NG on both feed cost and enteric methane emissions. We conducted a case study with 675 cows from the University of Wisconsin's Arlington Agricultural Research Station. Diets were formulated using the farm's actual feed ingredients and prices and were constrained according to current dairy cattle feeding guidelines. Without NG, optimization alone reduced feed cost by $2.52/cow per day (32%) under the cost-minimization objective, and reduced methane emissions by 57 g/cow per day (12%) under the methane-minimization objective, relative to the on-farm diet used during the study period. Under a dual-objective approach that simultaneously considered feed cost and enteric methane emissions, the model identified a pragmatic compromise solution. By placing greater emphasis on economic performance to reflect the current US production context, methane emissions decreased by 55 g/cow per day compared with the cost-minimization scenario while maintaining similarly low feed costs. Nutritional grouping did not consistently outperform the optimized single-group diet, but it modestly improved the nontarget outcome (lower methane when minimizing cost and lower cost when minimizing methane). These findings indicate that well-optimized rations using existing feeds can deliver meaningful economic and environmental benefits, and NG offers an additional layer of management refinement that may not only reduce the feed cost as suggested by literature, but also offer complementary benefits when methane is considered as a secondary performance dimension.

## Highlights

• Linear optimization reduced feed cost by 32% and methane by 12% when each was targeted.
• Nutritional grouping modestly improved the nontarget trait in single-objective diets.
• Dual-objective optimization balanced cost savings with methane reduction.
• Improvements came from small tweaks to existing rations, not major reformulation.

## Graphical Abstract

**Figure:**
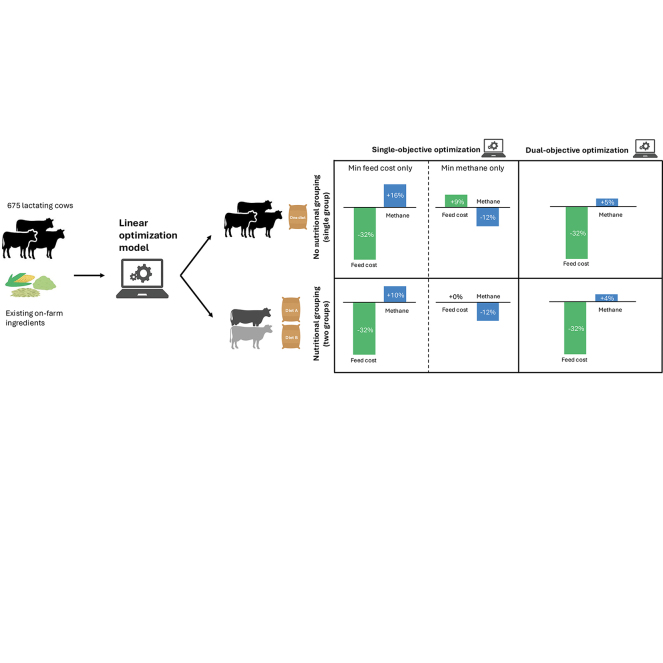
**Summary:** We developed an open-source linear optimization model to assess whether nutritional grouping (NG) of lactating dairy cows can reduce feed cost and enteric methane production beyond optimized single-group feeding. Using data from 675 cows at the University of Wisconsin-Madison Arlington Research Farm and the farm's existing feed ingredients, we compared an optimized single-group diet with 2-group strategies based on milk yield, days in milk, or net energy for lactation. When optimized separately, the single-group diet reduced feed cost by 32% (cost objective) and methane by 12% (methane objective) relative to the base diet. Under a dual-objective formulation, methane decreased by 10% relative to the cost-minimization solution while maintaining near-minimum feed cost. Across criteria, NG did not markedly outperform the optimized single-group diet but modestly improved nontarget outcomes and nutrient precision. These results suggest that small, data-driven adjustments to existing diets can enhance economic and environmental sustainability.

Nutritional grouping (**NG**) is a herd management strategy that involves separating lactating cows into groups based on their specific nutritional requirements. Each group receives a tailored TMR formulated according to their production stage (e.g., parity, DIM), performance level (e.g., milk yield), or health and reproduction status, in order to optimize nutrient intake and enhance herd productivity and efficiency (Cabrera and Kalantari, 2016). Since the 1970s, extensive research has primarily investigated this practice for its benefits in production efficiency and economic performance (Wilk et al., 1978; Wu et al., 2019).

However, most existing NG studies have focused on grouping algorithms aimed at minimizing the within-group variance in nutritional requirements or maximizing herd income over feed cost (**IOFC**). For example, McGilliard et al. (1983), Kalantari et al. (2016), and Barrientos-Blanco et al. (2020) employed clustering algorithms to group cows with similar nutritional needs, thereby reducing within-group variance. Cabrera et al. (2012) and Wu et al. (2019) used iterative search methods to identify optimal grouping strategies for maximizing IOFC. Despite advances in grouping algorithms, these studies have primarily focused on high-level nutritional requirements without investigating the implications of group-specific feed formulation using diverse on-farm feed ingredients.

Beyond economic benefits, NG, as a precision feed management strategy, also has the potential to improve nutrient use efficiency (Chase and Fortina, 2023). Simulation work by St-Pierre and Thraen (1999) suggested that NG could increase milk production and reduce nitrogen excretion, whereas observational studies have reported mixed results. Some studies observed limited improvements in nutrient use efficiency from NG (Jonker et al., 2002; Powell et al., 2006), whereas others reported enhanced nutrient use efficiency (Arriaga et al., 2009). However, the potential role of NG in mitigating enteric methane has received much less attention. Most recent methane reduction efforts have focused on whole-herd dietary interventions such as altering grain content or adding feed additives such as ionophores (e.g., monensin), saponins, tannins, 3-nitrooxypropanol, or *Asparagopsis taxiformis* (Hristov et al., 2022). To date, methane mitigation through NG and group-level ration refinement remains largely unexplored.

To ensure a fair comparison, both NG and non-NG scenarios were optimized rather than contrasted against a nonoptimized baseline. Therefore, our objective was to develop an open-source linear optimization model capable of minimizing feed cost, enteric methane emissions, or performing dual-objective optimization, under scenarios with and without NG. We also present a case study using data from the University of Wisconsin's Arlington Agricultural Research Station (Arlington, WI) to illustrate how dietary adjustments differ across objectives and grouping strategies. We hypothesized that NG could facilitate more targeted dietary strategies, potentially enabling additional reductions in feed cost, enteric methane emissions, or both, compared with a single-group approach.

We modified the linear optimization model in Gong et al. (2025) to formulate diets for lactating dairy cows under 3 objective settings: (1) minimizing feed cost (Equation 1), (2) minimizing enteric methane emissions (Equation 2), or (3) a duo-objective minimization (Equation 3). The enteric methane emissions were estimated based on equation 14-1a in NASEM (2021). For the dual-objective case, we applied a weighted sum approach in which the 2 objectives were combined into a single function by assigning relative weights (Marler and Arora, 2004). We used a 1:1 weight ratio between feed cost ($/cow per day) and enteric methane emissions (kg/cow per day) in their native units. Because feed costs are typically $5 to $10/cow per day whereas methane is ∼0.5 kg/cow per day, this formulation places greater emphasis on economic performance while still allowing methane mitigation to influence the solution, consistent with practical farm priorities.

Objective functions:

(1) minimize feed cost; [1]
(2) minimize CH_4_; [2]
(3) minimize (feed cost + CH_4_). [3]

This study targets commercial confined dairy systems where cows are fed TMR. We assume that the feed ingredients are homogenized into a TMR that is fully consumed by the cows to which it is delivered. In this paper, “feed cost” refers to total ration cost based on ingredient prices, including valuations for forages. We did not separately estimate purchased-only feed cost, which may differ depending on farm-specific accounting of homegrown feeds.

To evaluate the performance of our model, we conducted a case study using data from the University of Wisconsin's Arlington Agricultural Research Station (Arlington, WI). The dataset (test day data in January 2025) included records for 675 lactating cows and contained information on cow ID, parity, DIM, milk yield (kg/d), fat (%), and protein (%). In this study, milk yield and composition were treated as a cow-level input and were held constant across all scenarios. The cows' BW were estimated using the approach described in Giordano et al. (2012). Dry matter intake and NEL were estimated based on equations 2-1 and 3-14c in NASEM (2021). The lactating cow diet used on the farm during the study period is referred to as the base diet in this study and served as the comparison baseline. Its detailed formulation was described in Pupo et al. (2024; Table 1, covariate diet). Our study formulated rations using only feed ingredients available in the base diet. The ingredient prices were obtained from the farm manager, except for corn silage, alfalfa silage, cottonseed, and high-moisture corn grain, which were sourced from the literature (Li et al., 2022) and online article (Blonde, 2018). Nutrient composition values of feed ingredients were primarily sourced from Table 19-1 in NASEM (2021), except for corn silage, alfalfa silage, and high-moisture corn, whose values were obtained from laboratory analyses conducted by Vita Plus (Madison, WI).

Our model formulates diets based on specified DMI and NEL requirements, with DMI and NEL constrained to match target values within ± 1%. Following dairy industry convention, the TMR for each group was formulated to meet the nutritional requirements of the 83rd percentile cow using lead factors (Stallings and McGilliard, 1984). The CP, NDF, starch, and fat were maintained within the NASEM (2021) recommended ranges: CP (15%–18% DM), NDF (25%–33% DM), starch (22%–30% DM), and fat (0%–7% DM). In this context, CP was used as a practical safeguard consistent with guideline ranges rather than as a biological requirement target. Maximum inclusion rates of each ingredient were set to ensure that no single feed component was included at an unreasonably high level. We constrained the proportion of DM from silages in the diet to 40% to 60% to align with the typical forage inclusion levels in Wisconsin. Additionally, the ratio of corn silage to alfalfa silage was constrained between 1:1 and 2:1, reflecting local land availability and regional practices for alfalfa use in Wisconsin.

Based on previous literature, we selected 3 grouping criteria for this study: milk yield, DIM, and NEL. Milk yield and DIM are the most commonly used grouping strategies in commercial dairy operations (Jonker et al., 2002; Contreras-Govea et al., 2015), whereas some studies have shown that grouping by NEL requirements can lead to greater economic efficiency (Kalantari et al., 2016; Wu et al., 2019).

The model was developed using Python (Python Software Foundation; https://www.python.org/) and solved using the Gurobi Optimizer (Gurobi Optimization LLC; https://www.gurobi.com/). The complete source code is open-source and available on GitHub at https://github.com/YijingGong/Diet-optimization-and-nutritional-grouping. The repository enables users to minimize feed cost, enteric methane production, or both, with or without applying grouping strategies. Users can select between 2 methane prediction equations, choose among 3 grouping criteria (milk yield, DIM, or NEL), and specify the number of groups (2 or 3). Detailed usage instructions and examples are provided in the repository's README file.

To characterize the study herd and examine potential impacts of NG, we calculated descriptive statistics for all lactating cows under both a single-group scenario and several hypothetical 2-group NG strategies based on milk yield, DIM, or NEL (Table 1). In each grouping scenario, cows were ranked based on the grouping criterion and then divided (in silico) into 2 equal-sized groups. The resulting statistics demonstrate clear differences between subgroups, indicating a potential for precise feeding using NG.

**Table 1:** Descriptive statistics (mean ± SD) for lactating cows from the University of Wisconsin Arlington Agricultural Research Station (Arlington, WI) with no nutritional grouping (NG; 675 cows as a single group), or with 2-group strategies based on either milk yield, DIM, or NEL (groups of 338 and 339 cows, respectively)

NG strategy		Group	Parity	DIM (d)	Milk yield (kg/d)	Fat (%)	Protein (%)	BW^1^ (kg)	DMI^1^ (kg/d)	NEL^1^ (Mcal/kg DM)
No NG		—	2.74 ± 1.68	158 ± 97	42 ± 10	4.27 ± 1.04	3.47 ± 0.45	692 ± 51	27.1 ± 2.8	1.67 ± 0.14
Milk yield		Low	2.22 ± 1.58	183 ± 107	34 ± 6	4.64 ± 0.89	3.64 ± 0.52	677 ± 61	25.3 ± 2.5	1.61 ± 0.15
		High	3.26 ± 1.62	133 ± 78	50 ± 7	3.90 ± 1.04	3.29 ± 0.29	706 ± 32	28.8 ± 1.9	1.73 ± 0.11
DIM		Low	2.75 ± 1.77	79 ± 40	45 ± 11	4.19 ± 1.10	3.32 ± 0.35	677 ± 49	27.0 ± 3.5	1.74 ± 0.13
		High	2.73 ± 1.60	237 ± 69	39 ± 8	4.35 ± 0.97	3.61 ± 0.50	706 ± 49	27.2 ± 1.9	1.60 ± 0.12
NEL		Low	2.78 ± 1.58	207 ± 92	37 ± 8	4.07 ± 0.84	3.57 ± 0.51	706 ± 49	26.4 ± 2.2	1.57 ± 0.10
		High	2.70 ± 1.78	110 ± 76	46 ± 10	4.47 ± 1.17	3.36 ± 0.37	678 ± 49	27.7 ± 3.2	1.78 ± 0.09

1 Body weights were estimated using the approach described in Giordano et al. (2012); DMI and NEL were estimated based on Equations 2-1 and 3-14c in NASEM (2021), respectively.

When the model was configured to minimize feed cost, optimizing the diet for the single-group (no NG) scenario reduced costs from $7.98/cow per day (base diet) to $5.46/cow per day, a 32% reduction (Table 2). The optimized ration met CP requirements near the lower constraint, reflecting the use of less expensive, lower-protein feeds. Nutritional grouping did not provide additional cost reduction ($5.44, $5.48, and $5.47/cow per day across the 3 NG criteria). This contrasts with previous studies, with Wu et al. (2019) reporting increases in IOFC of $40 to $48/cow per year and Kalantari et al. (2016) observing an increase of $39/cow per year with 2-group clustering. The larger benefits in those studies likely reflected their reliance on CP and NEL prices alone, whereas our model incorporated individual feed ingredient prices and additional nutrient constraints (NDF, starch, and fat). Moreover, the diet formulation was intentionally limited to the 10 feed ingredients already used on the farm to align with the current ration and reflect actual ingredient availability, which reduced optimization flexibility. With a broader range of ingredients, NG might provide greater cost savings. Methane emissions increased from the base diet (486 g/cow per day) to the cost-minimized single-group diet (565 g/cow per day), primarily due to the high inclusion of soybean hulls (rich in NDF and digestible NDF [**dNDF**], Figure 1). Nutritional grouping strategies partially offset this increase, lowering emissions to ∼535 g/cow per day. This reduction was driven by lower DMI in the low-energy group and lower dNDF in the high-energy group.

**Table 2:** Feed cost, enteric methane production, DMI, and the diet nutrient composition for lactating cows from the University of Wisconsin Arlington Agricultural Research Station (Arlington, WI) under 3 optimization objectives (minimize feed cost, minimize methane, and dual-objective optimization), with no nutritional grouping (no NG; 675 cows as a single group) or with 2-group NG strategies based on either milk yield, DIM, or NEL (groups of 338 and 339 cows, respectively)

Item	Base diet	Minimize cost							Minimize methane							Minimize both^1^						
		No NG	Milk yield		DIM		NEL		No NG	Milk yield		DIM		NEL		No NG	Milk yield		DIM		NEL	
			Low	High	Low	High	Low	High		Low	High	Low	High	Low	High		Low	High	Low	High	Low	High
Feed cost ($/cow per day)	7.98	5.46	4.94	5.94	5.72	5.25	5.02	5.92	8.72	8.31	8.41	7.43	8.79	8.64	7.41	5.46	4.96	5.94	5.72	5.27	5.03	5.92
Average	—	—			5.44			5.48			5.47	—	8.36	8.11	7.98			—	5.45	5.49	5.48	
Methane production^2^ (g/cow per day)	486	565	519	554	507	559	543	524	429	391	467	426	431	415	443	510	461	554	507	505	521	524
Average	—	—	536	533	534	—	429	429	429	—	507	506	523									
DMI (kg/cow per day)	26.8	26.4	24.3	28.2	26.4	26.1	25.4	27.1	26.0	24.3	27.6	25.8	26.1	25.4	26.6	26.3	24.3	28.2	26.4	26.1	25.4	27.1
DM (% as fed)	52.5	56.5	56.5	56.5	56.5	56.5	56.6	56.5	58.6	58.7	58.6	58.6	58.7	56.4	58.6	56.5	56.5	56.5	56.5	56.6	56.6	56.5
NEL (Mcal/kg)	1.79	1.81	1.79	1.84	1.86	1.75	1.70	1.87	1.84	1.79	1.87	1.90	1.75	1.70	1.90	1.82	1.79	1.84	1.86	1.75	1.70	1.87
CP (% DM)	17.2	14.9	15.2	15.2	16.3	15.2	15.2	16.7	20.2	20.2	20.2	20.2	20.2	20.2	20.2	15.0	15.2	15.2	16.3	15.2	15.2	16.6
NDF (% DM)	27.7	32.8	33.3	32.2	30.6	33.3	33.3	30.2	25.3	25.3	25.3	25.3	25.3	25.3	25.3	32.9	33.3	32.2	30.6	33.3	33.3	30.2
dNDF (%DM)	9.15	14.0	14.3	11.4	10.4	14.3	14.3	10.3	7.1	6.7	7.2	7.3	6.6	6.5	7.3	11.7	11.7	11.4	10.4	11.9	13.3	10.3
Starch (% DM)	26.1	29.8	30.3	29.7	29.7	30.3	30.3	29.7	30.3	30.3	30.3	30.3	30.3	30.3	30.3	29.9	30.3	29.7	29.7	30.3	30.3	29.7
Fat (% DM)	6.0	3.0	3.0	4.6	4.7	3.0	2.9	4.7	7.1	7.1	7.1	7.1	7.1	7.1	7.1	4.7	4.9	4.6	4.7	4.7	3.6	4.7

1 Duo-objective optimization using weighted sum approach (Marler and Arora, 2004).

2 The enteric methane emissions were estimated using Equation 14-1a in NASEM (2021).

**Figure 1:**
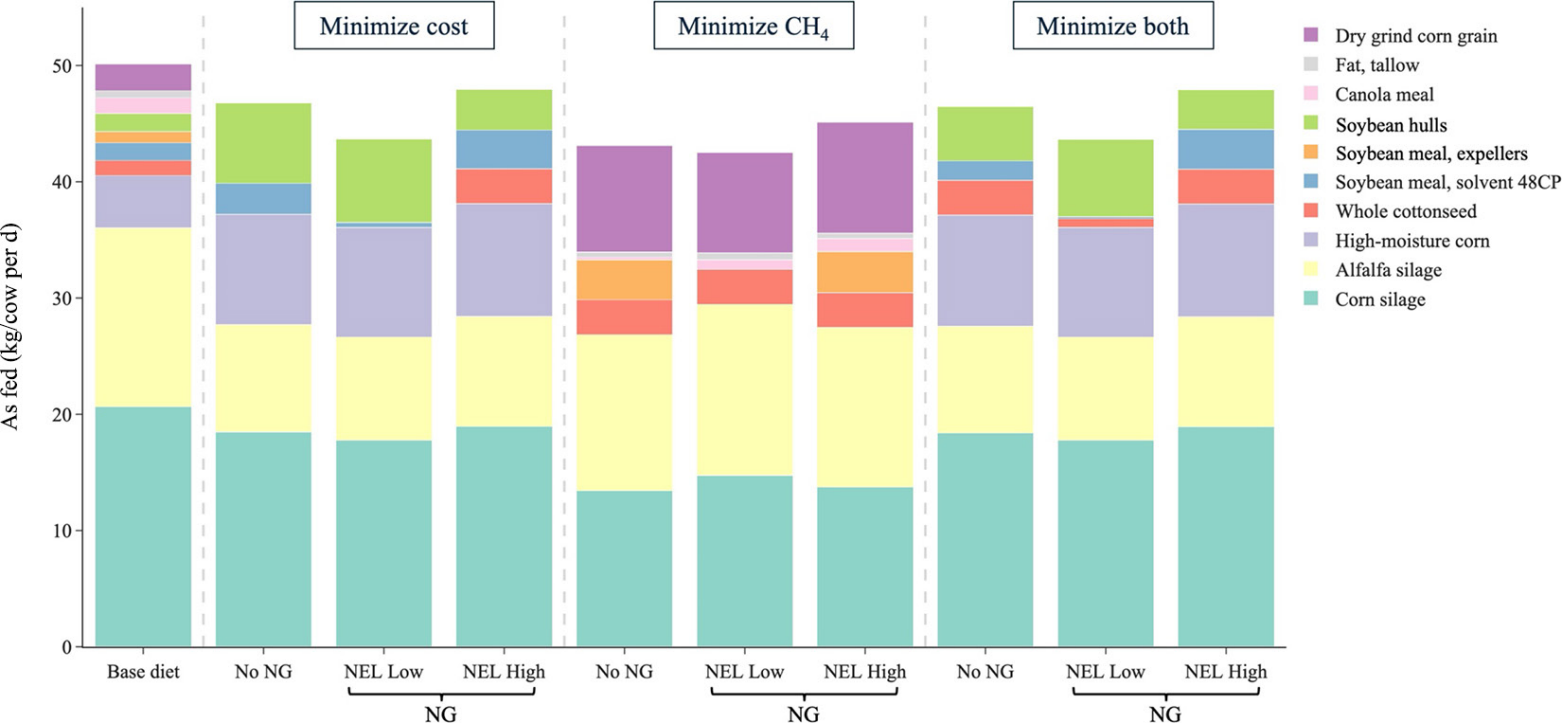
Diet ingredient composition (as fed, kg/cow per day) for lactating cows at the University of Wisconsin–Madison Arlington Agricultural Research Station (Arlington, WI) under the base diet and optimized diets with no nutritional grouping (no NG) or 2-group nutritional grouping (NG) based on NEL, evaluated under 3 different optimization objectives. Supplement ingredients included at less than 1 kg/cow per day were omitted for clarity.

When the objective shifted to minimizing enteric methane emissions, the model reduced methane output to 429 g/cow per day across all strategies, representing a 12% decrease relative to the base diet. However, this reduction came with higher feed costs: $8.72/cow per day for the single-group scenario, and $8.36, $8.11, and $7.98/cow per day for each of the 3 NG scenarios, respectively (Table 2). This trade-off has been well documented in the literature (Moraes et al., 2012; Gong et al., 2025). The methane-optimized diets had higher CP, starch, and fat content, along with lower DMI and dNDF, aligning with established nutritional strategies for methane mitigation (Hristov et al., 2022). However, the elevated CP content (20.2%) raises concerns regarding nitrogen use efficiency and the possibility of increased nitrogen excretion, indicating a need for further investigation in future work. Nutritional grouping strategies did not further reduce methane emissions compared with the single-group approach. Although dividing cows into multiple groups allows more precise feeding of NEL by meeting the 83rd percentile requirement for each group, the key nutrients influencing enteric methane—fat and NDF—were constrained within the same allowable ranges across all groups. Consequently, the single-group diet had already maximized fat and minimized NDF, leaving little room for NG to provide additional environmental benefits. This suggests NG could potentially offer benefits if more granular, animal-specific requirements for nutrients influencing methane production (e.g., NDF and fat), beyond DMI and energy alone, were incorporated. Nevertheless, we observed decreases of $0.36 to 0.74/cow per day in feed cost with NG compared with the optimized no-NG scenario. Under NEL-based grouping, the feed cost was $7.98/cow per day, the lowest among all methane-minimization scenarios. This matched the base diet cost while achieving a 12% reduction in methane emissions relative to the base diet.

When performing duo-objective optimization, the model achieved a reasonable balance between economic and environmental goals. Average feed costs ($5.46–$5.49/cow per day) were almost identical to those achieved under cost minimization alone ($5.46–$5.48/cow per day). Methane emissions were reduced to 506 to 523 g/cow per day compared with 533 to 565 g/cow per day in the cost-minimization scenario; nevertheless, they remained higher than the base diet (486 g/cow per day). This outcome arises because dual-objective optimization does not guarantee simultaneous improvement in both objectives relative to a given baseline, particularly when the objectives are inherently competing, as is the case for feed cost and enteric methane emissions. Such trade-offs are well documented in the literature (Moraes et al., 2012; Gong et al., 2025). In this study, we intentionally placed greater emphasis on economic performance to reflect the prevailing US production context, under which methane reductions are typically pursued only when they do not increase feed cost. The resulting diets reflected a compromise, with CP levels comparable to the cost-minimizing scenario and exhibiting modest shifts in dNDF and fat toward values observed under methane minimization. Under this dual-objective approach, NG did not yield clear additional benefits in terms of feed cost or methane outcomes under all 3 grouping criteria.

Figure 1 illustrates the diet ingredient composition (as fed, kg/cow per day) for the base diet and optimized diets with no NG or NEL-based NG strategies across different optimization objectives. Across all scenarios, corn silage and alfalfa silage remained the dominant ingredients, as the model constrained silages to provide 40% to 60% of dietary DM. However, their proportions varied with the optimization objective and grouping. When the objective was to minimize feed cost, the number of ingredients used declined from 10 in the base diet to 5 cost-efficient options, with dry ground corn grain, fat tallow, canola meal, and soybean meal expellers excluded. Both silages were reduced relative to the base diet, particularly alfalfa silage, given its higher cost. In contrast, high-moisture corn grain and soybean hulls increased markedly. High-moisture corn grain provided an economical source of energy with a moderate amount of CP, whereas soybean hulls provided a low-cost supply of CP and NDF. Soybean meal (solvent extracted, 48% CP) was retained as an efficient supplier of both NEL and CP. In the high-NEL group, the model further increased the use of soybean meal (solvent extracted, 48% CP) and added whole cottonseed to meet the greater energy requirements. This diet formula minimized costs while fully meeting nutrient constraints, with adjustments that represent refinements rather than fundamental changes to the base diet. The main shifts involved greater use of soybean hulls and high-moisture corn, which substituted for a portion of the silages and other more expensive ingredients. These adjustments are consistent with practical feeding strategies reported in the literature. Studies have shown that partial substitution of forage with soybean hulls or high-moisture corn can maintain, or even improve, milk and fat yield, provided that overall NDF levels and particle size are properly managed (Valadares Filho et al., 2000; Miron et al., 2003; Pupo et al., 2024).

Scenarios targeting minimum enteric methane emissions led to more pronounced shifts in diet formulation. Corn silage was reduced, likely due to its relatively high NDF and dNDF content, and high-moisture corn was eliminated. In its place, diets included more dry ground corn grain, which is high in starch, as well as whole cottonseed, which is high in fat. In the no-NG group and high-NEL NG group, greater amounts of soybean meal (expellers) were also incorporated to increase NEL and fat levels. These adjustments align with established nutritional strategies for mitigating methane emissions, which emphasize higher dietary starch and fat while lowering NDF (Knapp et al., 2014; Eugène et al., 2021). It is also notable that in both the low- and high-NEL groups, the model consistently selected the maximum allowable inclusion of alfalfa silage, constrained to equal the amount of corn silage to reflect practical land availability for alfalfa silage production in Wisconsin. The model's tendency to favor alfalfa silage over corn silage is consistent with other diet optimization studies (Moraes et al., 2012; Gong et al., 2025). This preference likely reflects the lower NDF content of alfalfa silage relative to corn silage. In addition, its high CP and low starch content complement the high-starch, lower-CP dry corn grain, enabling the ration to maximize starch inclusion while still meeting protein requirements.

In the duo-objective scenario, the model minimized a weighted sum of feed cost and enteric methane emissions. Because the economic objective was prioritized, the resulting ingredient composition closely resembled the cost-minimization case, with similar amounts of corn silage, alfalfa silage, and high-moisture corn. However, soybean hulls and soybean meal (expellers) were used in lower amounts, whereas whole cottonseed was increased. Although the overall formula remained close to the cost-minimization diet, the duo-objective optimization still reduced methane emissions by 55 g/cow per day in the single-group scenario and by 11 to 29 g/cow per day under 3 NG scenarios. These results show that relatively small adjustments to diet composition can simultaneously support economic efficiency and environmental sustainability on dairy farms.

This simulation study demonstrates the strong potential and practical applicability of a linear optimization model for dairy diet formulation using only feed ingredients already available on-farm. The optimized diets did not differ drastically from current feeding practices, yet substantially improved performance by reducing feed costs or enteric methane emissions when each objective was targeted individually. Optimizing the single-group diet decreased feed cost by 32% or methane emissions by 12% relative to the base diet, depending on the objective. The dual-objective approach provided a practical compromise, achieving the lowest possible feed cost while reducing methane emissions by 10% compared with cost minimization alone, consistent with the prevailing US production context where methane mitigation is pursued only when it does not adversely affect economic performance. Alternative objective weightings and their broader implications were discussed in our previous work (Gong et al., 2025). Nutritional grouping did not yield dramatic additional reductions in cost or methane within the targeted goal, differing from prior studies that reported stronger economic benefits. This discrepancy may stem from our use of individual feed ingredient prices, whereas other studies relied on nutrient prices. Nevertheless, NG provided complementary gains: methane decreased by 29 to 32 g/cow per day when optimizing for cost only, and feed cost declined by $0.36 to $0.74/cow per day when optimizing for methane only, relative to the single-group results. Under dual-objective optimization, NG offered limited additional benefits for both goals. Beyond these targets, NG enhanced nutrient precision by improving alignment between cow requirements and dietary crude protein levels at similar costs. A limitation of this study is the assumption that milk production was not responsive to small diet changes. To support this assumption, we constrained optimized diets to remain nutritionally comparable to the base ration by requiring DMI and NEL to stay within ± 1% of targets and by retaining the same ingredient set under practical maximum inclusion limits. However, future work could incorporate responsive production models and iterative optimization and assess IOFC under both total and purchased feed cost definitions. In addition, although CP was constrained within guideline ranges as a pragmatic nutritional safeguard, this remains a simplified proxy relative to metabolizable protein– or amino acid–based rationing. The elevated CP content under the methane-minimization objective underlines the need for future work to evaluate nitrogen excretion risks. Overall, this study shows that modest, optimization-guided adjustments to existing diets can meaningfully enhance both economic and environmental sustainability in dairy nutrition management.
